# Is there an impact of autoimmune rheumatological diseases on cutaneous toxicity in breast cancer adjuvant radiotherapy? A mono-institutional experience

**DOI:** 10.1007/s12282-025-01791-7

**Published:** 2025-10-13

**Authors:** Giulia de Pasquale, Valeria Donatiello, Marco Lucarelli, Rosario Bonelli, Marta Di Nicola, Annamaria Porreca, Annamaria Vinciguerra, Marianna Nuzzo, Consuelo Rosa, Andrea D’Aviero, Domenico Genovesi

**Affiliations:** 1 Department of Radiation Oncology, “S.S. Annunziata” Chieti Hospital, Chieti, Italy; 2 Department of Medical, Oral and Biotechnological Sciences, “G. D’Annunzio” University of Chieti, Chieti, Italy; 3 Laboratory of Biostatistics, Department of Medical, Oral and Biotechnological Sciences, “G. D’Annunzio” University of Chieti, Chieti, Italy

**Keywords:** Breast cancer, Rheumatological disorders, Radiotherapy

## Abstract

**Aims:**

Autoimmune rheumatological diseases (ARDs) have historically represented an absolute or relative contraindication for radiotherapy (RT) due to increased RT-related toxicity and the potential exacerbation of rheumatologic disease. ARDs are more frequent in females (F:M 4:1). Breast cancer (BC) is the most common malignancy, accounting alone for 31% of female cancers. This study compared acute and late cutaneous toxicity in ARDs and non-ARDs population undergoing adjuvant breast RT.

**Methods:**

Data of patients with BC and ARDs treated between 2013 and 2023 were retrospectively reviewed. The ARDs group was compared with a control group in a 1:2 ratio, homogeneous by age, type of treatment, RT total doses and fractionations, and target volumes’ prescription. Acute and late toxicity were recorded using RTOG scales.

**Results:**

We included 44 women with ARDs (median age 61 years) and 88 woman (median age 62 years) as control group. In ARDs group, the most used RT schedules were conventional fractionation (72.7%), while hypofractionation schedule (40–44 Gy) was administered in 12 patients (27.3%). In the control group, 64 patients (72.7%) received RT with conventional fractionation and 24 patients (27.3%) hypofractionation (40–44 Gy). Overall acute skin toxicity rate was 80.4% in the control group vs 86.4% in the ARDs group (*p* = 0.681). Specifically, G2 toxicity was 22.0% in the control group vs 31% in the ARDs group, while G3 acute toxicity was 2.3% in both groups. Overall late skin toxicity was 21.6% in the control group vs 27.3% in ARDs group (*p* = 0.067). Statistically significant difference was observed in late G2 toxicity with a 0% rate in the control group vs 6.8% in the experimental group (*p* = 0.035), respectively.

**Conclusions:**

ARDs do not seem to represent an absolute or relative contraindication in BC RT in terms of acute and late cutaneous toxicity. Hypofractionated schedule showed less toxicities in both group and, particularly, in ARDs group.

## Introduction

Breast cancer (BC) is the most common malignancy in women, accounting alone for 31% of female cancers [1]. After conservative surgery, adjuvant radiotherapy (RT) represents the gold standard in treating BC [2]. Furthermore, in the presence of selected risk factors, irradiation can also concern the lymph-node stations and the breast wall post-mastectomy [3–6].

Autoimmune rheumatological diseases (ARDs) are chronic and heterogeneous clinical disorders [7]. The spectrum of autoimmune disease includes several disorders. Some of these are more common, such as rheumatoid arthritis (RA), fibromyalgia, systemic lupus erythematosus (SLE), and scleroderma. In contrast, others are less frequent, such as Sjogren’s disease, Bechet’s disease, dermatomyositis, psoriatic arthritis, polymyalgia rheumatica, and spondylitis. The ARDs are also more frequent in females versus males (F:M 4:1) [7, 8]. These chronic conditions historically have represented an absolute or relative contraindication to RT due to increased RT-related acute and late cutaneous toxicity and to the possible exacerbation of the rheumatological disease [9, 10].

While case reports published in the 1980s–1990s showed an increase in RT-related toxicity in ARD patients, more recent studies did not report an increased risk of acute or late toxicities between ARDs and non-ARDs groups with modern RT technology and dose prescriptions [11, 12]. The most important data about this aspect are related to the CONTRAD study [13], a 2019 meta-analysis that evaluated 621 patients undergoing radiotherapy from 1970 to 2018 and presenting as comorbidities ARDs and inflammatory bowel disease. In the above-mentioned, 417 patients with rheumatological diseases were included, including 245 patients with RA, 55 with SLE, and 44 with Scleroderma. The study showed that ARDs and inflammatory bowel disease were not absolute contraindications to radiotherapy. A 10–15% of any G3 toxicity and less than 5% of G4 were reported.

Previously, the 2003 Phan comparative study too [14] also showed no significant differences in acute and late toxicity between patients with ARDs and the control group, except for a higher incidence of radiation complications in patients with scleroderma.

The overcoming of these contraindications is likely due to the technological implementation of RT techniques and to a better clinical management of the patient; in particular, the arrival of intensity-modulated radiation therapy (IMRT) in the mid-to-late 2000s has reduced the RT toxicity [15].

In 2020, Purswani et al. reported a case–control study on 91 patients with rheumatic autoimmune disease and BC [16]. Among the ARDs considered, the study focused on RA (21%), SLE (8%), and Sjogren’s (8%). The study, which focused on a wide range of cosmesis-related toxicities, did not show significant differences in late cutaneous toxicity in ARD patients compared to the normal population. The evaluation of incidence rates in relation to the fractionation schedule also revealed no differences. In fact, in the conventional fractionation, the incidence of CTCAE grade 2–3 acute skin toxicity versus the control group did not show statistically significant differences. In contrast, an increase in late skin toxicity occurred in the ARDs group, although without statistical significance. Regarding patients treated with hypofractionated RT, no difference in grade 2–3 acute or late cutaneous toxicity was found comparing the ARDs setting versus the control group.

In 2021, Yoon et al. analyzed patients with different cancers and ARD comorbidities, reporting toxicity rates associated with three dose-fractionated RT schedules: conventional fractionation (CF; 2 Gy per fraction), moderate hypofractionation (MH; > 2 Gy to < 5 Gy), and ultra-hypofractionation (UH; 5 Gy per fraction) [17]. The study, which evaluated 197 patients, included different subgroups of ARDs such as RA (74 patients), SLE (34 patients), and Scleroderma (8 patients). The overall incidence of global acute and late G3–G4 toxicity, according to the CTCAE scale, was less than 10% in all dose-fractionation groups.

However, some ARDs have been slightly investigated by studies on RT, particularly Fibromyalgia. Indeed, in one of the largest matched-control studies about ARDs, Phan et al. reported only 3 patients with Fibromyalgia (8%). In contrast, other ARDs were more represented, such as SLE (55%), Scleroderma (5%), Sjogren’s (8%), and Polymyalgia rheumatica (8%) [14]. In a comparative analysis between non-ARD patients and the control group, no significant difference was found in the incidence of global acute (G2 49% vs 58% or G3 7% vs 7%) and late (G1 3% vs 7%; G2 7% vs 3%; G3 7% vs 7%) toxicity (RTOG scale), with a higher incidence of radiation toxicity found in patients with scleroderma.

Our case–control study aimed to evaluate RT treatment compliance in terms of acute and late cutaneous toxicity in BC patients with ARDs. We also investigated the possible impact of RT fractionation schedules in ARDs.

## Materials and methods

In a whole cohort of BC patients, the ARDs experimental group was compared with a control group without ARDs, homogeneous for age, sex, adjuvant RT doses and fractionation, target volumes prescriptions, and tumor stages. Table 1 reports Median and Interquartile Range [Q1; Q3] for the main characteristics of the analyzed groups.

**Table 1:** Median and interquartile range [Q1; Q3] in both groups

	Controls	ARDs	*p* value
	*n* = 88	*n* = 44	
Age (years)	61.5 [51.8; 67.0]	62.0 [52.0; 68.5]	0.484
Total dose	5000 [4256; 5000]	5000 [5000; 5000]	0.594
Fractionate dose	200 [200; 266]	200 [200; 200]	0.196

The collection of patients with ARDs was based on the clinical characteristics, serological test, and/or histopathological diagnosis performed by the referring rheumatologists of the individual patients. The evidence of active ARDs was investigated before starting the RT course and during the treatment in terms of disease-related symptoms and the use of antirheumatic and/or steroid drugs.

Acute and late RT toxicity were reported according to the Radiation Therapy Oncology Group (RTOG) 17 criteria of common toxicity; the timing is defined as within 90 days post-RT for acute toxicity and over 3 months for late toxicity. Adverse events were graded from 0 to 4 (0: no toxicity; 4: worse toxicity). The incidence and possible differences in RT toxicity among the different subgroups of patients and the different fractionation schedules were also investigated.

### Statistical analysis

Descriptive statistics were expressed as median (first; third) quartile. The Chi-square test was used to test the association between categorical variables and 95% confidence interval (95% CI) were determinate to evaluate a precision of the ratios. The Mann–Whitney *U* test was used to determine statistically significant differences between the control and ARD groups. All tests used are two-tailed, and the alpha error level was chosen to be 0.05. All analyses were performed with the R environment (version 4.1; http://www.r-project.org/).

Propensity score matching (PSM) was used to account for matching between groups. Propensity scores were estimated using a logistic regression model that considered patient age, adjuvant RT doses, and fractionation. Subsequently, the ARDs group were compared in a 1:2 ratio without replacement with the control group using a propensity score with a caliper of 0.25 standard deviation of the logit of the propensity score. SMDs were also calculated in the matched sample to compare characteristics between groups. An SMD < 0.15 was used as an indicator of adequate matching.

## Results

### ARDs group

We retrospectively analyzed 47 patients affected by BC and ARDs, submitted to adjuvant RT between 2013 and 2023. The patients' ages ranged between 41 and 78 years, with a median age of 61 (52.8; 68.5 years). 44/47 patients were evaluated for the study aims. Three patients were lost in follow-up, so they were not included for lacking of data.

In our study, we registered the following ARDs: 15 RA (34.1%), 12 Fibromyalgia (27.3%), 3 SLE (6.8%), 3 Scleroderma (6.8%), 2 Spondylitis (4.5%), 3 Sjögren's syndrome (6.8%), 3 Polymyalgia Rheumatica (6.8%), 1 Psoriatic Arthritis (2.3%), 1 Dermatomyositis (2.3%), and 1 Bechet's disease (2.3%).

All ARD patients treated conservatively had BC in the I–II stages (early stage). In particular, T staging was 13.6% T in situ, 2.3% T1a, 25% T1b, 45.5% T1c, and 13.6% Stage II. 42 patients (95.5%) underwent RT after a conservative surgery, while only 2 patients (4.5%) underwent RT after a mastectomy surgery.

The most used RT schedule for ARD patients was a conventional fractionation (50–54 Gy/2.0 Gy for fraction) in 32 patients (72.7%). Hypofractionation (40–44 Gy/2.66–2.67 Gy for fraction) was administered in 12 patients (27.3%). The fractionation schedule in the hypofractionation group was 2.67 Gy per day. A tumor bed boost of 10 Gy, 2 Gy/die was prescribed in 32 (72.7%) patients.

Regarding target volume definition, residual breast only (84.1%) was irradiated in 37 patients. Breast and the supraclavicular lymph-node region in 4 patients (9.1%), breast wall only in 1 patient (2.3%), and breast wall and the supraclavicular region (4.5%) in 2 patients. Regarding systemic therapies, chemotherapy was administered as primary neoadjuvant therapy in 15.9% of cases (7 patients) and adjuvant pre-RT therapy in 18.2% (8 patients). In 65.9% (29 patients), chemotherapy was not administered. In 77.3% of cases (34 patients), adjuvant hormone therapy was prescribed.

All patients completed the RT course: one patient, affected by Bechet Syndrome, temporally discontinued RT because of systemic disease exacerbation with complete recovery, reporting G1 acute toxicity; another patient, affected by Scleroderma disease, temporally discontinued RT course for a skin G3 acute toxicity. Both patients received conventional fractionation (50 Gy/2.0 Gy for fraction), followed by a tumor bed boost of 10 Gy; both patients stopped the RT course for about 2 weeks; then, they regularly completed the RT course without other complications and through biological recalculation of the total dose.

The median follow-up was 4.8 years (0.4; 7.1 years). After RT, none of the ARD patients showed disease exacerbation or needed further therapy for their autoimmune disease status.

### Control group

The control group included 88 patients who received adjuvant Radiotherapy between 2014 and 2023. The median age of the control group was 62, ranging from 32 to 81 (51.8; 67 years).

All patients in the control group had BC staging between stages I and II (early). In particular, the T stage was 14.7% T in situ, 5.7% T1a, 14.7% T1b, 37.5% T1c, 21.6% stage II, and only 3.4% stage III.

About target volume irradiation, most of the patients received RT to the breast only, 72 patients (81.8%); 10 patients (11.4%) had RT to the breast and supraclavicular lymph nodes; 6 patients received RT after mastectomy: breast wall was irradiated in only one patient (1.1%) while in the other 5 patients (5.7%), the supraclavicular lymph nodes were irradiated with the breast wall. Regarding systemic therapies, 22 (25.0%) received primary neoadjuvant chemotherapy, and 13 (14.8%) received adjuvant chemotherapy. Adjuvant hormone therapy was prescribed in 71 patients (80.7%).

64 patients (72.7%) received conventional treatment (50–54 Gy), while 24 (27.3%) patients received the hypofractionation schedule (40–44 Gy). In 64 (72.7%) patients, a 10 Gy, 2 Gy/die tumor bed boost was prescribed. All patients completed the RT course without discontinuation.

The median follow-up was 3.7 years (2.60; 5.31 years).

### ARDs group vs control group: toxicity comparison results

The 44 patients of the ARDs experimental group were matched 1:2 with 88 patients in the control group analyzed retrospectively in our database. Matching was attempted according to age, irradiated target volumes, total dose, fractionation, boost dose, and stage to compare any toxicity recorded in the two groups.

No substantial difference in the incidence of acute skin toxicity was observed between the two groups. All patients in both groups experienced acute toxicity. The overall acute skin toxicity (RTOG scale) recorded is similar in both groups (80.4% vs 86.4% in the control and ARDs groups, respectively; *p* = 0.681). Comparing the different grades of toxicity, G1 toxicity was 55.7% (49 patients) in the control group and 52.3% (23 patients) in the ARDs group (*p* = 0.853). For G2 toxicity, 22.7% was reported in the control group (20 patients) versus 31.8% (14 patients) in ARDs group (*p* = 0.360). The G3 acute toxicity was 2.3% in both groups: two patients in the control group and one in ARDs group (*p* = 1.00). No G4 toxicity was reported (Table 2).

**Table 2:** Controls vs ARDs for the acute and late cutaneous toxicity, according to RTOG scale

	All patients	Controls	ARDs	*p* value
	*N* = 132	*n* = 88	*n* = 44	
Acute toxicity (G1)	72 (54.5% [45.7, 63.2])	49 (55.7% [45.3, 66.1])	23 (52.3% [37.5, 67.0])	0.853
Acute toxicity (G2)	34 (25.8% [18.5, 34.1])	20 (22.7% [14.0, 31.5])	14 (31.8% [18.1, 45.6])	0.360
Acute toxicity (G3)	3 (2.3% [0.4, 6.5])	2 (2.3% [0.3, 8.0])	1 (2.3% [0.0, 12.0])	1.000
Late toxicity (G1)	28 (21.2% [14.6, 29.2])	19 (21.6% [13.5, 31.6])	9 (20.5% [9.8, 35.3]))	1.000
Late toxicity (G2)	3 (2.3% [0.5, 6.5])	0 (0.0%)	3 (6.8% [1.4, 18.7])	0.035
Late toxicity (G3)	0 (0.0%)	0 (0.0%)	0 (0.0%)	–

The *p* value derived from the Chi-squared test [95% CI]

Overall, late skin toxicity was 21.6% in the control group and 27.3% in the ARDs group (19 vs 12 patients; *p* = 0.067). In the control group, we observed only 26.1% (19 patients) of G1 late cutaneous toxicity versus 20.5% (9 patients) in the ARDs group (*p* = 1.00). We observed statistically significance regarding to G2 late skin toxicity: 0 (0%) patients in the control group versus 6.8% (3 patients) in ARDs group (*p* = 0.035). No severe G3–G4 late toxicity was reported in either group (Table 2).

In the comparison between the 2 different schedules (conventional vs hypofractionated), as shown in Table 3, the control group showed a more evident association for the acute G2 toxicity between the two types of treatment schedules. In fact, we observed an acute skin G2 toxicity in 31.2% of patients who received conventional fractionation CF vs 0.0% in hypofractionation HF, with statistical significance (*p* = 0.005). Overall, in both group, the hypofractionated treatment showed lower acute toxicity levels.

**Table 3:** Acute and late cutaneous toxicity distribution for treatments in ARDs and Control group

	ARDs			Controls		
	CF	HF	*p* value	CF	HF	*p* value
	*n* = 32	*n* = 12		*n* = 64	*n* = 24	
Acute toxicity (G1)	15 (46.9%)	8 (66.7%)	0.406	35 (54.7%)	14 (58.3%)	0.948
**Acute** **toxicity** **(G2)**	13 (40.6%)	1 (8.3%)	0.068	20 (31.2%)	0 (0.0%)	0.005
Acute toxicity (G3)	1 (3.1%)	0 (0.0%)	1.000	1 (1.6%)	1 (4.2%)	0.473
Late toxicity **(**G1)	8 (25.0%)	1 (8.3%)	0.405	18 (28.1%)	1 (4.2%)	0.032
Late toxicity **(**G2)	2 (6.2%)	1 (8.3%)	1.000	0 (0.0%)	0 (0.0%)	–
Late toxicity (G3)	0 (0.0%)	0 (0.0%)	–	0 (0.0%)	0 (0.0%)	–

The *p* value results from the Chi-squared test

*CF* conventional fractionation, *HF* hypofractionation

### ARDs group: toxicity comparison results in the different subgroups

Among the different ARDs, we observed the highest number of acute toxicities in Fibromyalgia, where, out of a total of 12 patients, 5 patients developed a G1 acute toxicity and 6 patients developed a G2 acute toxicity. In Rheumatoid Arthritis, out of a total of 15 patients, we registered G1 skin acute toxicity in 7 patients and a G2 skin acute toxicity in 4 patients.

Regarding late toxicity, we observed the highest number of late toxicities in Rheumatoid Arthritis, where, out of a total of 15 patients, 3 patients developed a G1 late toxicity.

Tables 4 and 5 contain all the toxicities data reported in each different ARDs subgroups.

**Table 4:** Acute cutaneous toxicity distribution in autoimmune rheumatologic diseases (ARDs)

Pathology	Acute toxicity G1			Acute toxicity G2			Acute toxicity G3		
	No	Yes	*p* value	No	Yes	*p* value	No	Yes	*p* value
	*n* = 21	*n* = 23		*n* = 30	*n* = 14		*n* = 43	*n* = 1	
Scleroderma	1 (4.8%)	2 (8.7%)	0.848	3 (10.0%)	0 (0.00%)	0.360	2 (4.6%)	1 (100%)	0.386
Rheumatoid Art	8 (38.1%)	7 (30.4%)		11 (36.7%)	4 (28.6%)		15 (34.9%)	0 (0.0%)	
Dermatomyositis	1 (4.8%)	0 (0.0%)		0 (0.0%)	1 (7.1%)		1 (2.3%)	0 (0.0%)	
SLE	2 (9.5%)	1 (4.3%)		1 (3.3%)	2 (14.3%)		3 (6.9%)	0 (0.0%)	
Sjogren	1 (4.8%)	2 (8.7%)		3 (10.0%)	0 (0.0%)		3 (6.9%)	0 (0.0%)	
Fibromyalgia	7 (33.3%)	5 (21.7%)		6 (20.0%)	6 (42.9%)		12 (27.9%)	0 (0.0%)	
Spondylitis	0 (0.0%)	2 (8.7%)		2 (6.7%)	0 (0.0%)		2 (4.6%)	0 (0.0%)	
Psoriatic Art.	0 (0.0%)	1 (4.3%)		1 (3.3%)	0 (0.0%)		1 (2.3%)	0 (0.0%)	
Bechet	0 (0.0%)	1 (4.3%)		1 (3.3%)	0 (0.0%)		1 (2.3%)	0 (0.0%)	
Polymyalgia Rh.	1 (4.8%)	2 (8.7%)		2 (6.7%)	1 (7.1%)		3 (6.9%)	0 (0.0%)	

The *p* value derived from the Chi-squared test

*Rheumatoid Art.* rheumatoid arthritis, *SLE* systemic lupus erythematosus, *Sjogren* Sjogren’s disease, *Psoriatic Art.* psoriatic arthritis, *Bechet* Bechet’s disease, *Polymyalgia Rh.* polymyalgia rheumatica

**Table 5:** Late cutaneous toxicity distribution in autoimmune rheumatologic diseases (ARDs)

Pathology	Late toxicity G1			Late toxicity G2			Late toxicity G3		
	No	Yes	*p* value	No	Yes	*p* value	No	Yes	*p* value
	*n* = 35	*n* = 9		*n* = 41	*n* = 3		*n* = 44	*n* = 0	
Scleroderma	2 (5.71%)	1 (11.1%)	1.000	2 (4.88%)	1 (33.3%)	0.306	3 (6.82%)	0 (0.00%)	–
Rheumatoid Art.	12 (34.3%)	3 (33.3%)		15 (36.6%)	0 (0.00%)		15 (34.1%)	0 (0.00%)	
Dermatomyositis	1 (2.86%)	0 (0.00%)		1 (2.44%)	0 (0.00%)		1 (2.27%)	0 (0.00%)	
SLE	2 (5.71%)	1 (11.1%)		3 (7.32%)	0 (0.00%)		3 (6.82%)	0 (0.00%)	
Sjogren	3 (8.57%)	0 (0.00%)		2 (4.88%)	1 (33.3%)		3 (6.82%)	0 (0.00%)	
Fibromyalgia	9 (25.7%)	3 (33.3%)		11 (26.8%)	1 (33.3%)		12 (27.3%)	0 (0.00%)	
Spondylitis	2 (5.71%)	0 (0.00%)		2 (4.88%)	0 (0.00%)		2 (4.55%)	0 (0.00%)	
Psoriatic Art.	1 (2.86%)	0 (0.00%)		1 (2.44%)	0 (0.00%)		1 (2.27%)	0 (0.00%)	
Bechet	1 (2.86%)	0 (0.00%)		1 (2.44%)	0 (0.00%)		1 (2.27%)	0 (0.00%)	
Polymyalgia Rh	2 (5.71%)	1 (11.1%)		3 (7.32%)	0 (0.00%)		3 (6.82%)	0 (0.00%)	

The *p* value derived from the Chi-squared test

*Rheumatoid Art.* rheumatoid arthritis, *SLE* systemic lupus erythematosus, *Sjogren* Sjogren’s disease, *Psoriatic Art.* psoriatic arthritis, *Bechet* Bechet’s disease, *Polymyalgia Rh*. polymyalgia rheumatica

### Propensity score matching

**Fig. 1:**
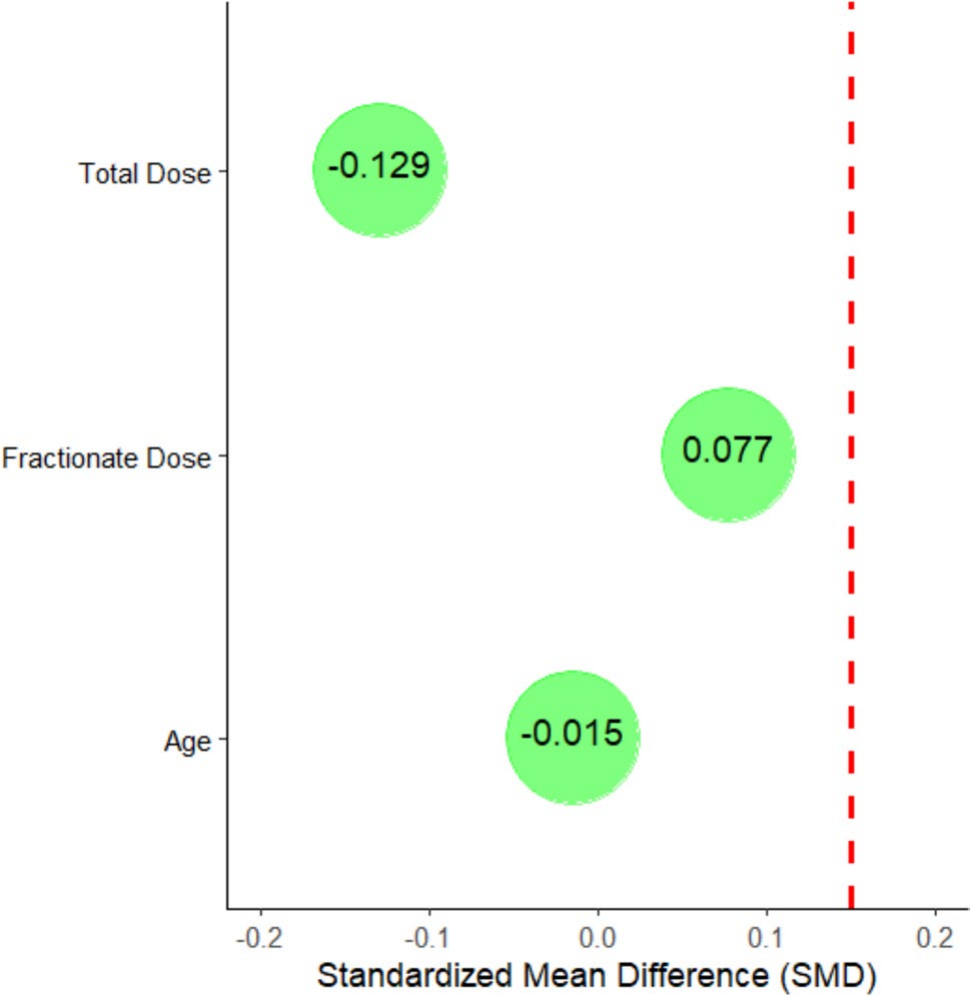
Standardized mean differences (SMD) for selected variables. The SMD values indicate a strong balance between groups for age (− 0.015), total dose (− 0.129), and dose per fraction (0.077), demonstrating the effectiveness of the matching process

Figure 1 reports the SMD for Age, Total Dose, and Fractionate Dose. The results of the Standardized Mean Difference (SMD) for the selected variables show the following insights: for age, the SMD is − 0.015, which is very close to zero, indicating an excellent balance between the groups. In terms of total dose, the SMD is − 0.129, falling below the threshold of 0.15, suggesting an acceptable level of balance. Finally, the SMD for dose per fraction is 0.077, which also reflects a good balance, as it remains below 0.1. Overall, these values indicate that the matching process has been effective in achieving a well-balanced distribution of the groups for these variables.

## Discussion

The presented study aimed to evaluate the incidence of acute and late cutaneous toxicity in ARDs patients that we observed in our Radiation Centre, to understand their toxicities and if the ARDs could be or not a contraindication for RT in BC patients. The incidence of skin toxicity was further investigated among the different subgroups of ARDs, and with the possible correlation with RT fractionation schemes (CF and HF). The data of ARDs patients were compared with a retrospectively identified control group with the same characteristics regarding age, type of treatment, dose administered, irradiation site, and presence of boosts. This was performed to assess a central objective of our study, to detect the presence of any differences in cutaneous toxicity between the two groups; in particular, an increase in cutaneous toxicity incidence in the ARDs group that might have justified a contraindication to RT.

Regarding the focus on the hypothesis that patients with ARDs could have a higher risk of toxicity from RT or whether they have access to this treatment with an acceptable level of risk, we reported acute G2/G3 skin toxicity is 34.1% in the ARDs group vs 25% in the control group, while late G2/G3 skin toxicity was in only 6.8% (3 patients) of the ARDs group. No late G2/G3 skin toxicity was reported in our control group. Although these data may suggest an increased risk of G2/G3 skin acute toxicity, they were not statistically significant in our study; thus, they do not justify excluding BC patients with rheumatic diseases from adjuvant RT.

These results are in line with the study of Shaikh et al., who conducted a meta-analysis in 2021: ten studies were included, with 4028 patients (ARDs: 406, control: 3622), to assess the risk of RT toxicity in patients with ARDs compared with controls [18]. They concluded that the absolute risk of serious toxicity is relatively low and does not justify stopping or reducing potentially life-saving treatments. The analysis of Parvez et al. defined a subgroup of patients with ARDs involving the breast who underwent RT; in these patients, an acute total G2/G3 toxicity rate of 21.5% was reported vs 15.7% of the group controls (OR = 1.92, 95% CI = 0.99–3.74; *p* = 0.05). Late total G2/G3 toxicity was 14.7% in the rheumatology group vs. 4.4% in the control group (OR = 3.51).

The meta-analysis and systematic review by Lin et al. in 2019, focusing on contraindications to RT, found similar results [13]. Among the 18 articles screened, 10 included ARD patients’ data (*n* = 417). The incidence of G3 toxicity in ARD patients (95% confidence interval) was 11.7% (5.4–19.6%) and 6.1% (1.4–12.6%) for acute and late toxicity, respectively. Also, these data showing < 5% risk of G4 toxicity and < 1% risk for G5 toxicity indicate that life-threatening consequences (G4 toxicity) and death (G5 toxicity) as direct results of RT are minimal in this patient population. Therefore, the researchers concluded that ARDs are not absolute contraindications to RT and our results confirmed this statement for ARDs breast cancer patients.

Recent studies have focused on assessing acute and late toxicity differences based on different fractionation schemes [16, 17]. Hypofractionation would seem to reduce the toxicity rates, mainly late complications, and may, therefore, be more beneficial in patients with ARDs.

Among these studies, the retrospective cohort study by Yoon et al. analyzed 197 adult patients cohort with cancer and ARDs, of which 48 had BC [17]. Three dose fractionation schedules were used: conventional fractionation (CF: 2 Gy per fraction), moderate hypofractionation (MH: > 2 Gy to < 5 Gy per fraction), and ultra-hypofractionation (UH: 5 Gy per fraction). Although radiobiological principles suggest that higher RT doses per fraction increase the risk of reactive tissue damage, MH radiotherapy and UH radiotherapy were associated with a lower likelihood of developing late toxic effects. Moreover, they observed that, on univariate analysis, UH radiotherapy was also associated with reduced odds of acute toxic effects compared with CF radiotherapy (OR = 0.14; 95% CI = 0.05–0.35; *p* < 0.001).

In 2021, also Purswany et al. investigated specifically breast conservation in women with autoimmune disease and the role of active autoimmune disease and hypofractionation on acute and late toxicity in a case-controlled series [16]. Among patients treated with hypofractionated RT, there was no difference in acute or late grade 2/3 toxicity between cases and controls (acute: 13.1% in cases vs. 11.5% in controls; *p* > 0.9); (late: 11.9% in cases vs. 13.1% in controls: *p* > 0.9). The good/excellent clinician-rated cosmesis rates were similar between groups (92.9% in cases vs. 98.9% in controls; *n* = 142).

Our statistical analysis report the differences in skin toxicity rates concerning the two treatment schedules. Regarding the ARD group, we analyzed two different fractionations: conventional (total dose: 50–54 Gy) in 72.7% of patients (32 patients) and hypofractionation (total dose: 40–44 Gy) in 27.3% of patients (12 patients). The analysis showed similar toxicity values between the conventional and hypofractionated treatment: in both fractionations, and there were no substantial differences in toxicity; G3 acute toxicity and G2–G3 late toxicity were similar (2 cases in CF and 1 case in HF, *p* value = 1.00). However, less acute G2 toxicity was observed in ARD patients who received hypofractionation: acute G2 toxicity is 40.6% (13 patients) in CF vs 8.3% (1 patient) in HF (*p* = 0.068).

The control group was also analyzed by fractionation schedule and we found statistically significant data on acute G2 toxicity, which was 31.2% (20 patients) in CF vs 0% in HF (*p* = 0.05). Moreover, we observed a similar trend even for late G1 toxicity with 28.1% (18 patients) in CF vs 4.8% (1 patient) in HF group (*p* = 0.032). These results suggest that hypofractionated radiotherapy may be most appropriate in women with autoimmune disease and BC, as it has also reported by Wu et al. whose findings demonstrated similar acute skin adverse reactions between CF and HF and they indicated that hypofractionated radiotherapy offers comparable tolerance, equivalent curative effect, convenience, and economic benefits, supporting its clinical promotion [19].

Regarding subgrouping ARDs patients in their different clinical manifestations, patients with RA and Fibromyalgia constituted the largest samples in our study.

Starting from RA, in a retrospective matched-pairs study, Dong et al. [20] compared the skin toxicity and cosmesis in 40 women with RA to 117 controls without RA who received RT for BC. In this comparison, there was no significant difference in the rates of G2 acute toxicity (25.0 vs. 13.7%, OR 2.1, CI 0.91–4.9) or G2 late toxicity (7.5 vs. 4.3%, OR 1.8, CI 0.48–6.8). Mean cosmesis was between good and excellent in both groups of patients, although women with RA were less likely to achieve excellent cosmesis compared to their matched pairs (OR 0.35, CI 0.15–0.84). Researchers have concluded that in women with RA, radiation for BC was well tolerated without significantly increased cutaneous toxicity.

Moreover, Fiorica et al., in their retrospective observational study on RA patients where breast cancer constituted 28% of the study group, observed that RT was well tolerated with low rates of both acute and late toxicity and it was not associated with an increased risk of articular flares [21].

In our study, RA was the most common type of ARD. The G1 and G2 acute skin toxicity rates in the RA group were 46.7% (7 patients) and 26.7% (4 patients), respectively. We found only a G1 rate of 20% (3 patients) and no G2 toxicity for the late cutaneous toxicity rate. No G3 toxicity rates were reported in our population of women with RA. None of the patients had a re-activation of the disease. These results are similar to the previously mentioned RA and BC RT studies.

Regarding to Fibromyalgia, some studies have focused on the quality of life of patients in relation to cancer. The analysis of Akkaya et al. showed that a Fibromyalgia diagnosis impacts negatively on pain and fatigue in BC patients [22]. Eyigor et al., for example, explored the prevalence of Fibromyalgia in 122 patients with different cancer types, but only eight of them had BC. Their data seem to indicate a higher incidence of Fibromyalgia in the oncological population [23].

However, very few studies have concentrated on RT treatment of Fibromyalgia patients and specifically on breast irradiation; 3 patients with Fibromyalgia were mentioned only in the case series of the study by Phan et al., in which there were no significant differences in the incidence of acute and late skin toxicity from RT [14].

Looking deeply at our results in the Fibromyalgia population, which, for our knowledge, at the moment, it is one of the largest RT-treated group of breast cancer patients with Fibromyalgia in literature, there was an increase in mild-to-moderate acute skin toxicity. Out of a total of 12 patients, 5 patients developed a G1 acute toxicity rate and 6 patients developed a G2 acute toxicity rate. The lowest rates of skin late toxicity were G1 25% (3 patients) and G2 8.3% (1 patient). For both acute and late skin toxicity, no G3 toxicity rate was found. In general, although related to a small sample, our data suggest that Fibromyalgia does not appear to be a contraindication to conservative treatment of BC with RT.

About the other ARD subgroups, some studies show a higher risk of radiation skin toxicity in patients with Scleroderma and SLE. However, the number of patients in many of these studies with this diagnosis is limited. Chen et al. analyzed a cohort of 36 women, 4 of whom were diagnosed with Scleroderma, observing a higher incidence of acute and late skin toxicity among patients with Scleroderma after breast RT (any acute: 50% vs. 0% in controls; any late: 75% vs. 0% in controls). This study includes cases from 1975 to 1998, before 2000, and the technological improvements in radiotherapy may explain this increased risk of toxicity. All these 36 patients were treated with conventional radiation therapy to a total medium dose of 64 Gy [24].

A more recent retrospective study conducted by Shah et al. in 2018 focused specifically on the toxicity of conservative BC treatment in women with Scleroderma [25]. This is the largest study of scleroderma patients with BC treated with RT. In these patients, significant acute skin toxicity (blistering, ulceration) from radiation was uncommon, while approximately 50% developed long-term radiation-induced cutaneous fibrosis that was localized to the field of radiation. Scleroderma cutaneous subtype, autoantibody status, and disease duration were not associated with a higher risk of radiation-induced skin thickening.

About SLE, Benk et al. [26] attempted to determine whether RT is denied to patients with SLE and whether it can cause excessive toxicity. They looked at 40 cases of cancer in 38 patients with SLE. Unaware of the SLE diagnosis, three radiotherapists were asked to review the patient's medical records. They recommended RT in 26 cases, but only 4 patients received RT. None of these patients developed any unusual skin toxicity. Breast irradiation was only given to 2 patients but would have been recommended for 8. It is interesting to see how, although the literature is evolving on this topic, there is a certain hesitation in treating patients with ARDs, so the fear of adverse effects leads to omitting radiation treatment for ARD patients unnecessarily.

In our data, we have a sample of only 3 patients with Scleroderma. Two patients developed acute G1 toxicity (66.7%), and one patient developed acute G3 toxicity (33.3%). No acute G2 toxicity was observed. As regards late skin toxicity, we have 1 patient who developed G1 (33.3%) and 1 patient who developed G2 toxicity (33.3%), while no cases of G3 late toxicity have been reported. Regarding the SLE, we have data from only 3 patients in our study. None of the 3 patients developed acute or late skin G3 toxicity. One patient developed acute G1 toxicity, and two patients developed acute G2 toxicity.

There are some limitations to the current study. The mono-institutional and retrospective design and the restricted sample examined may determine statistical limitations, although our group aligns with the number of other studies on the topic. Like previous publications, we were limited by the heterogeneity of ARD subtypes, resulting in a modest number of patients analyzed for each subtype. Our analysis was also limited to patients of Caucasian descent. There may have been bias during the selection of the control group or missing data that excluded patients eligible for the ARDs group.

In evaluating the acute and late skin toxicity grade, we considered the RTOG scale. However, to match the studies that used this different scale, we converted it to the Common Terminology Criteria for Adverse Events (CTCAE) scale. These two systems are the most used in RT and in oncology to evaluate skin toxicity in breast irradiation. We are aware that comparing the two scales for acute and late skin toxicity can imply differences that could define a different clinical impact. These two systems differ, because the RTOG scale uses different, mainly descriptive criteria to assess acute and late toxicity. It is used in clinical practice, because it is easy to record and consult. The CTCAE scale, on the other hand, is not only newer but also more complex, going so far as to subdivide 28 different categories of adverse events but does not distinguish between acute and late events. Therefore, when analyzing our retrospective data using the RTOG scale, we may have missed some clinical indicators of adverse events. However, overall, the scales are almost comparable in this context, especially in acute toxicity [27].

## Conclusion

The main aim of our study was to understand if oncological patients with rheumatological diseases can have therapeutic RT strategies comparable to those of the normal population in terms of toxicity occurrence and compliance.

In summary, despite a diagnosis of ARDs historically appearing to predispose patients to a risk of RT toxicity, treatment is generally well tolerated, with a very low incidence of severe acute or late skin toxicity. The low rate of mild and serious side effects, either in this study or in those compared, could indicate that RT complementary treatment for BC could be extended to include women with ARDs.

The experience in the literature about the RT treatment of rheumatology patients affected by BC is still very restricted. Considering the growing incidence of immune diseases and cancer, the interaction between RT and ARDs needs to be clearer to guarantee complete access to the best appropriate care for these patients, too. A prospective analysis also considering the possible impact of dosimetric parameters on toxicity rates, such as the correlation of bone marrow irradiation with ARDs exacerbation, has been designed to validate the presented data.

## Data Availability

The data presented in this study are available on request from the corresponding author.
